# Biofuel Ash Aging in Acidic Environment and Its Influence on Cd Immobilization

**DOI:** 10.3390/ijerph20054635

**Published:** 2023-03-06

**Authors:** Le Song, Feng Zhao, Haiyang Cui, Jingmin Wan, Hui Li

**Affiliations:** 1 Hebei and China Geological Survey key Laboratory of Groundwater Remediation, Institute of Hydrogeology and Environmental Geology, Chinese Academy of Geological Sciences, Shijiazhuang 050061, China; 2 School of Resources and Environmental Engineering, Hefei University of Technology, Hefei 230009, China; 3 Hebei Province Collaborative Innovation Center for Sustainable Utilization of Water Resources and Optimization of Industrial Structure, Hebei GEO University, Shijiazhuang 050031, China; 4 Hebei Geological Environment Monitoring Institute, Shijiazhuang 050021, China; 5 College of Home Economics, Hebei Normal University, Shijiazhuang 050024, China; 6 Shijiazhuang City Longquan Lake Garden Affairs Center, Shijiazhuang 050000, China

**Keywords:** biofuel ash, aging, acidic environment, influencing mechanism, Cd immobilization

## Abstract

Biofuel ash (BFA), which is the ash generated by biomass combustion in a biomass power plant, can be prepared as a heavy metal immobilizer and have a good immobilization effect on Cd in the soil environment of southern China, but the long-term effects of BFA on Cd immobilization remained unclear. Therefore, research about BFA aging and its influence on Cd immobilization was conducted in the paper. BFA was naturally aged into BFA-Natural aging (BFA-N) in the soil environment of southern China, and to simulate BFA-N, BFA was also artificially acid aged into BFA-Acid aging (BFA-A). The result indicated that BFA-A could partially simulate BFA-N in physicochemical properties. The Cd adsorption capacity of BFA reduced after natural aging and the decrease was more obvious in BFA-A according to $Q_{m}$ in Langmuir equation and $q_{e}$ from the pseudo-second-order kinetic model. The adsorption processes of BFA before and after aging were mainly controlled by chemical action rather than physical transport. The immobilization of Cd included adsorption and precipitation, and adsorption was the dominant factor; the precipitation proportion was only 12.3%, 18.8%, and 1.7% of BFA, BFA-N, and BFA-A, respectively. Compared with BFA, both BFA-N and BFA-A showed Ca loss, and BFA-A was more obvious than BFA-N. Ca content level was consistent with Cd adsorption level among BFA, BFA-N, and BFA-A. It could be inferred that the main immobilization mechanism of Cd by BFA before and after aging was consistent and closely related to Ca. However, the adsorption mechanism of electrostatic interaction, ion exchange, and hydroxyl complexation changed to varying degrees in BFA-N and BFA-A.

## 1. Introduction

Excessive heavy metal content exists in the southern farmland of China [1], in terms of soil pollution risk screening values of farming land [2]. For example, there is excessive Cd in part of the farmland in Yangtze river basin [3] and Pearl river basin [4]. However, from the perspective of the world, the Cd content of Chinese farmland is not too high on the whole, lower than that in European countries and America [5,6]. The accumulation of Cd in agricultural products is related to the total amount of Cd in soil, but also depends on the activity of Cd. For example, the total Cd content of soil in the UK is relatively high, but the Cd content of agricultural products rarely exceeds the standard; only 0.2% of wheat grain has a Cd content exceeding 0.2 mg/kg (Codex Alimentarius Commission Cd limit standard for wheat) [7]. In contrast, in the above major rice producing areas in the south of China [8], the exceeding standard rate of Cd in rice is high [9], and in some areas, the rate is as high as 60–70% [10,11], posing a threat to human health [12]. To ensure food safety [13], much research has been carried out on the immobilization of heavy metals in farmland soil [14,15]. Biofuel ash (BFA) is the ash generated by biomass combustion in a biomass power plant [16]. After the investigation and survey of multiple biomass power plants in southern China, what was found was that the heavy metal in BFA was volatile and enriched in the fly ash in the process of boiler combustion, and the heavy metal content in the bottom ash was less than 10% of the fly ash [17]. After screening, the bottom ash was prepared as a heavy metal immobilizer [18], and the adsorption capacity of Cd could reach more than 16 mg/g [19]. The content of available Cd in soil was significantly reduced by adding BFA of 1% soil dry weight in an in situ remediation experiment that was carried out in the farmland with severe Cd pollution in south China, and the Cd in rice was reduced by more than 70% [20].

The climate in south China is hot and humid, with a high vegetation coverage rate, strong decomposition of organic matter, and high leaching degree of topsoil [21]. Precipitation presents weak acidity due to the dissolution of CO_2_ in the air and the formation of carbonic acid [22,23]. The soil is affected by the long-term effects of geochemistry, ecological environment, and climate conditions, and the content of substitutable hydrogen and aluminum is high, so it tends to be acidic [24,25]. In addition to the above natural factors, NO_X_ and SO_2_ emitted by human activities dissolve in rainwater forming acid rain which falls into the soil and introduces certain NO_3_^−^ and SO_4_^2−^ [26,27], which makes the soil pH low and intensifies soil acidification degree [28,29]. The physical and chemical properties of BFA will be changed when it is applied in acidic soil in south China, that is, the aging of BFA may cause the release of minerals and affect the effect and mechanism of Cd immobilization [30]. The natural aging process is slow and time-consuming to monitor [31]. Artificial accelerated aging methods that simulate natural aging can significantly shorten the observation time, reducing the aging duration from years or months to days or hours [32]. In order to well simulate the process of acid aging, nitric acid (HNO_3_) and sulfuric acid (H_2_SO_4_) are usually selected to simulate acid rain [33].

Although BFA has achieved good results in the remediation of Cd-contaminated farmland in southern China, the aging of BFA and its long-term effects on Cd immobilization are still unclear. In this paper, BFA aging in an acidic soil environment in southern China will be studied to explore the change of physicochemical properties of BFA, and then to reveal the influence on the effect and mechanism of Cd immobilization. The artificial aging method is used to simulate natural aging of BFA in an acidic soil environment and its simulation effect is evaluated. The research will lay the foundation for the long-term application of BFA in acidic soil of southern China.

## 2. Materials and Methods

### 2.1. Materials Selection

Soil for the experiment was collected from the farmland of Daye City, Hubei Province, and the 0–20 cm topsoil was collected. The soil pH was 5.9. The heavy metal content of the soil was tested by the soil heavy metal analyzer Cadence (American XOS), and it did not exceed the screening value of soil pollution risk in agricultural land, meaning the pollution risk generally could be ignored in agricultural land environmental management of China [2]. The soil was ground through a 125 μm (120 mesh) sieve after air drying and set aside. BFA was obtained from the Jinzhou biomass power plant, Hebei Province, grinding and screening were in the size range of 250–500 μm (35–60 mesh) and 150 μm (100 mesh) particles.

### 2.2. Aging Methods

#### 2.2.1. Natural Aging

Ninety grams of BFA with a particle size of 250–500 μm (35–60 mesh) was added to 300 g soil, fully mixed in the culture box, and three groups were set in parallel. Natural aging was undertaken for 4 months and the mixture was placed in a 150 μm (100 mesh) screen. After ultrasonic cleaning, the surface of the screen was the aged BFA, which was then ground through a 150 μm (100 mesh) screen, and BFA-Natural aging (BFA-N) was obtained by freeze-drying. The pH of BFA-N was 7.8.

#### 2.2.2. Artificial Acid Aging

We diluted 70.54 mL of 98% concentrated sulfuric acid to 1000 mL with water to prepare 20% sulfuric acid. Then, 343.08 mL of 65% nitric acid was diluted to 1000 mL with water and prepared as 20% nitric acid. Then, 300 mL of 20% sulfuric acid and 100 mL of 20% nitric acid were added to the beaker and mixed. Finally, 5 g BFA, through 150 μm (100 mesh) particles, was added to the mixed acid solution; after 6 h of aging, the BFA-Acid aging (BFA-A) was obtained by freeze-drying [34,35]. The pH of BFA-A was 2.5.

### 2.3. Physical–Chemical Tests

X-ray fluorescence spectrograph Axios (Netherlands PANa-lytical, Almelo, The Netherlands), X-ray diffractometer D8-ADVANCE (German Brucker), Fourier transform infrared IS10 (American Nicolet), Surface Area Instrument NOVA 4000e (American Quantachrome), ZETA potentiometer Nano ZS (British Malwen Instruments), and Field emission scanning electron microscope SU8202 (Japanese Hitachi) were used to determine the physical and chemical properties of BFA, BFA-N, and BFA-A.

### 2.4. Adsorption Kinetic Experiment

BFAs (BFA, BFA-N, BFA-A) of 100 mg before and after aging were weighed, respectively, and placed in 50 mL centrifuge tubes; each group set up three repeats. Twenty milliliters of 200 mg/L Cd^2+^ reserve solution and 20 mL of 0.01 mol/L Ca^2+^ background solution were added to the centrifuge tubes. The centrifuge tubes were placed in a 25 °C constant temperature shaking incubator and samples were taken at 0 min, 5 min, 10 min, 15 min, 20 min, 30 min, 1 h, 2 h, 4 h, 6 h, 8 h, 12 h, and 24 h, respectively. After centrifugation and filtration, Cd^2+^ concentration of the supernatant was measured by ICP Avio500 (American PerkinElmer, Waltham, MA, USA).

### 2.5. Isothermal Adsorption Experiment

BFAs (BFA, BFA-N, BFA-A) of 100 mg before and after aging were weighed, respectively, and placed in 50 mL centrifuge tubes, with three replicates in each group. Forty milliliters of Cd^2+^ solution with concentrations of 5, 10, 20, 40, 60, 80, 100, and 160 mg/L were prepared by 200 mg/L Cd^2+^ reserve solution and 0.01 mol/L Ca^2+^ background solution in centrifuge tubes. The centrifuge tubes were placed in a constant temperature shaking incubator at 25 °C for 24 h. After centrifugation and filtration, Cd^2+^ concentration of the supernatant was measured by ICP Avio500 (American PerkinElmer).

### 2.6. Precipitation Experiment

Forty milliliters of Cd^2+^ solution with concentration of 40 mg/L ($C_{0}$) was prepared using 200 mg/L Cd^2+^ reserve solution and 0.01 mol/L Ca^2+^ background solution in centrifuge tubes. We used 1 mol/L HCl and NaOH solutions to adjust the pH according to the equilibrium solution in the isothermal adsorption experiment. The centrifuge tubes were placed in a constant temperature shaking incubator at 25 °C for 24 h. After centrifugation and filtration, Cd^2+^ concentration of supernatant was measured by ICP Avio500 (American PerkinElmer), expressed as $C_{24}$. The precipitation capacity ($Q_{p}$) was obtained by $Q_{p}$ = $40\;\mathrm{mL}*(C_{0}-C_{24})/100\;\mathrm{mg}$.

## 3. Results and Discussion

### 3.1. Changes in Physical and Chemical Properties of BFA induced by Aging

#### 3.1.1. Mineral Composition Change by XRD

**Figure 1:**
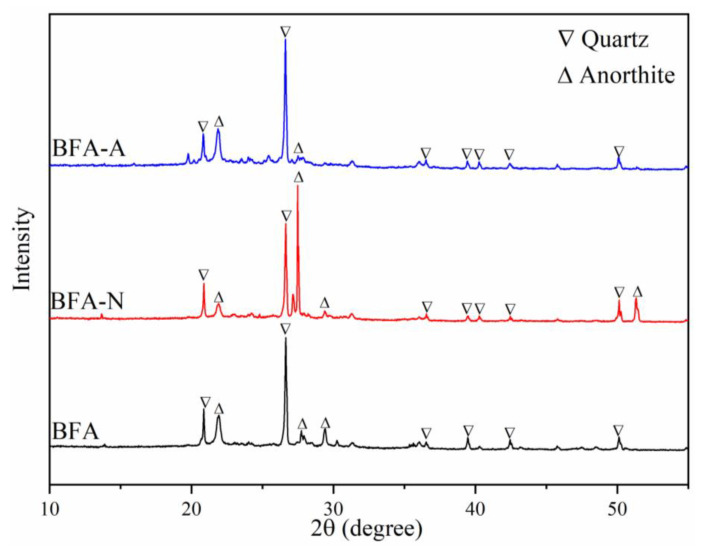
Mineral composition of BFA, BFA-N, and BFA-A.

The main elements of BFA, which were greater than 1%, were shown in Table 1. Combining the main element composition, the mineral morphology of BFA before and after aging was shown in Figure 1. BFA, BFA-N, and BFA-A all contained anorthite in addition to quartz. The operating temperature of the gasifier in the biomass power plant was between 800 and 1100 °C, and within the sintering temperature range. Qin [36] synthesized anorthite by solid phase reaction from lime and fly ash, which had consistent element composition with BFA. The difference in the mineral morphology of BFA before and after aging was that the anorthite crystal in BFA-N was more obvious, while it was relatively weakened in BFA-A. In addition, there was some amorphous morphology in the three samples.

#### 3.1.2. Functional Group Change by FTIR

**Figure 2:**
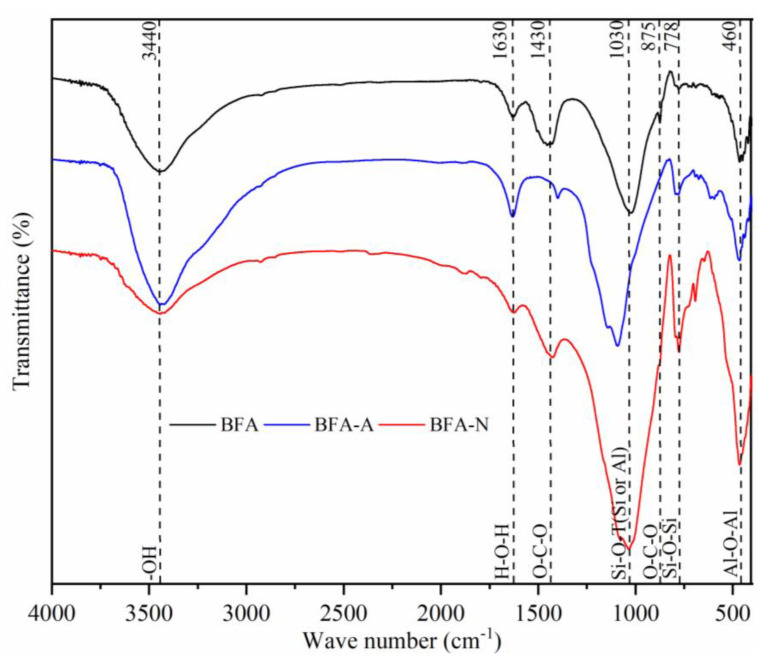
Functional groups of BFA, BFA-N, and BFA-A.

As shown in Figure 2, 3440 cm^−1^ was the stretching vibration peak of the hydroxyl group, and 1630 was the bending vibration peak of water. The peaks of BFA-A at both the above places were enhanced, indicating that the surface hydroxyl group was strengthened, while the peaks of BFA-N at both were weakened, indicating that the surface hydroxyl group was weakened. The antisymmetric vibration peak of O-C-O in CO_3_^2−^ was at 1430 cm^−1^, 875 cm^−1^ belonged to the out-of-plane bending vibration peak of O-C-O [37], the CO_3_^2−^ in BFA before aging was destroyed in BFA-A and BFA-N after aging in acidic conditions, and BFA-A was more obvious. The peak at 1030 cm^−1^ was related to the asymmetric stretching vibration of Si-O-T (T = Si or Al) of the tetrahedron inside the aluminosilicate [38]. BFA-A and BFA-N were enhanced here, and BFA-N was more obvious, BFA-A tended to move to a higher wave-number position, which is due to the fact that Si-O was shorter and stronger than Al-O, and the degree to which Al was replaced by Si increased. The stretching peak vibration of Si-O-Si in the silicon–oxygen tetrahedron oligomer was 778 cm^−1^, 460 cm^−1^ belonged to Al-O-Al stretching vibration, and the peak of BFA-A and BFA-N were enhanced here, indicating that there was silica–aluminate rearrangement during the aging process [39]. Combined with the analysis results of XRD, the aging process was conducive to the formation of amorphous geopolymers in BFA-N and BFA-A [40].

#### 3.1.3. Surface Morphology and Specific Surface Area Changes

**Figure 3:**
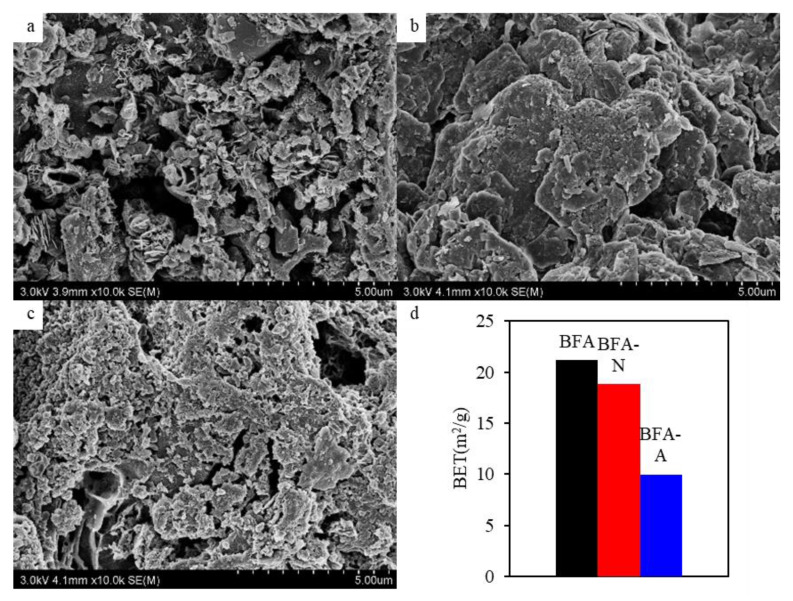
(**a**) SEM of BFA, (**b**) SEM of BFA-N, (**c**) SEM of BFA-A, and (**d**) BET.

The SEM of BFA, BFA-N, and BFA-A were shown in Figure 3a–c, respectively. BFA itself was loose and rough, with a lamellar overlapping and staggered morphology. Under the acidic environment of natural aging and simulated by artificial aging, the original pore structure was damaged to different degrees due to the change in material composition and structure. Under the influence of natural factors in the soil environment, some pores of BFA-N were filled and the surface was smoother. BFA-A was obtained by the artificial simulation aging method using concentrated acid, which exceeded natural conditions to accelerate aging. The accelerated aging increased oxygen-containing surface functional groups, such as hydroxyl, and reduced the overlapping and staggered efficiency of pore structure, resulting in the original channel collapsing, blocking, or disappearing [41]. The BET reduction of BFA-A was more obvious. The specific surface area shown in Figure 3d decreased from 21.16 m^2^/g for BFA to 18.80 m^2^/g for BFA-N and 9.96 m^2^/g for BFA-A, respectively.

#### 3.1.4. Zeta Potential Change

**Figure 4:**
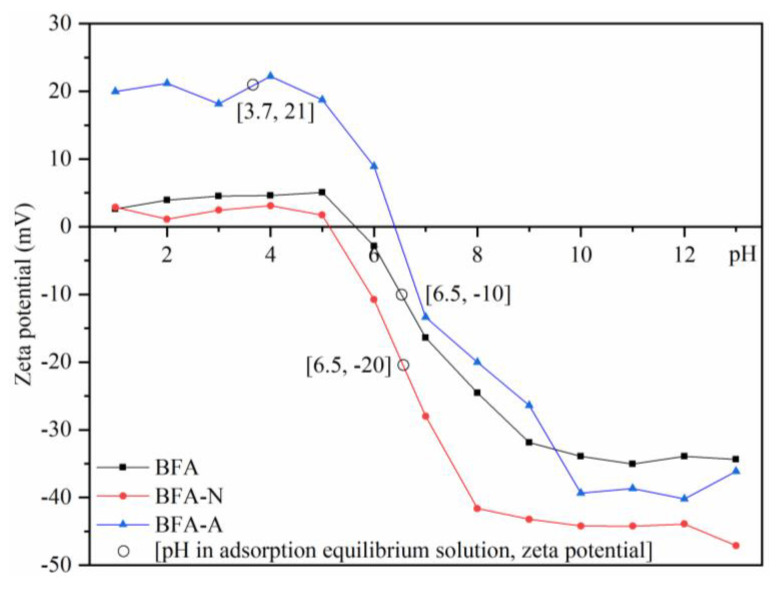
Zeta potentials of BFA, BFA-N, and BFA-A as a function of solution pH.

As shown in Figure 4, the pH_PZC_ of BFA was 5.6 where the surface charge was electrically neutral. After natural aging, the zero charge point of BFA-N moved to the left to 5.1, while after artificial acid aging, BFA-A moved to the right to 6.4. After aging, BFA-N and BFA-A showed a similar trend to BFA. When pH was 1–5, Zeta potential remained stable. When the pH is 5–9, BFA and BFA-N showed a decreasing trend, while when the pH was 5–10, BFA-A showed a decreasing trend, and the slopes of the Zeta potential reduction of all three were similar with the increase of pH. When the pH was 10–13, the three were stable, the solution system was mainly affected by precipitation, and the adsorption was weak. According to the solution pH of adsorption equilibrium, the Zeta potential values of BFA, BFA-N, and BFA-A in the system could correspond. It was worth noting that natural aging increased the negative charge on the surface of BFA, which was more conducive to the occurrence of electrostatic adsorption, while artificial acidic aging significantly increased the surface H^+^, which was still positively charged in the adsorption equilibrium system, which was not conducive to the adsorption of heavy metal cations.

#### 3.1.5. Comparison of Physicochemical Properties between BFA-N and BFA-A

**Figure 5:**
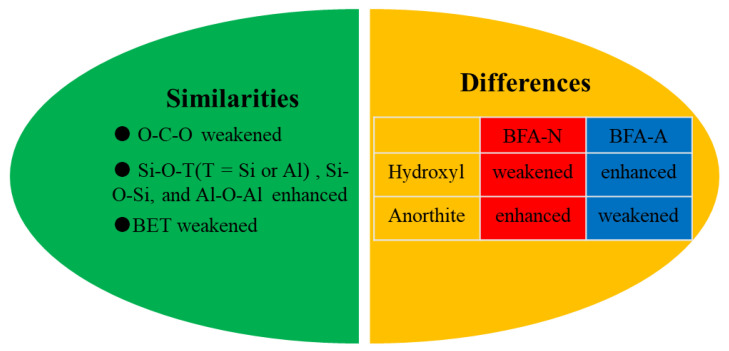
Similarities and differences in physicochemical properties’ change between BFA-N and BFA-A.

There were similarities and differences in physicochemical properties between BFA-N and BFA-A, that is, the artificial acid aging method could partially simulate natural aging. Compared with BFA, BFA-N and BFA-A both had the following changes: O-C-O weakened, Si-O-T (T = Si or Al), Si-O-Si, and Al-O-Al enhanced, and BET weakened. The differences were different trends of change in hydroxyl and anorthite phases as shown in Figure 5. Although artificial acid aging could simulate natural aging to a certain extent, BFA aging in the soil environment was a complex process involving physics, chemistry, and biology at the same time. In addition to the acidic factor considered in this study, oxygen, light, minerals, microorganisms, and other factors all had certain effects on aging. However, the effective response of artificial aging to natural aging in time still needed to be verified on the basis of representative natural monitoring data obtained from long-term series analysis.

### 3.2. Influence on Cd Immobilization Effect Induced by the Aging of BFA

#### 3.2.1. Adsorption Kinetic Experiment

Pseudo-first-order kinetic model:

(1) $$\ln\left(q_{e}-q_{t}\right)=\ln(q_{e})-k_{1}t$$

Pseudo-second-order kinetic model:

(2) $$\frac{t}{q_{t}}=\frac{1}{k_{2}q_{e}{}^{2}}+\frac{1}{q_{e}}t$$

In Equation (1), $q_{e}$ was the fitted value of equilibrium adsorption capacity, mg/g, $q_{t}$ was the adsorption capacity at time *t*, mg/g, $k_{1}$ was pseudo-first-order adsorption rate constant, 1/h. In Equation (2), $k_{2}$ was pseudo-second-order adsorption rate constant, g/mg·h [42]. 

**Figure 6:**
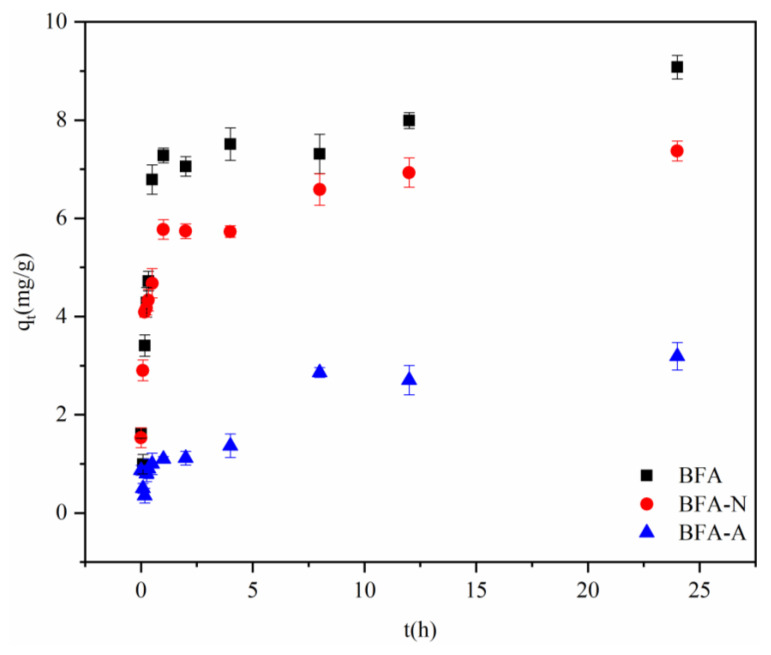
Absorption kinetic curves of Cd by BFA, BFA-N, and BFA-A.

As shown in Figure 6, the adsorptions of Cd by BFA, BFA-N, and BFA-A were rapid in the first hour, and then slowed down until adsorption equilibrium. The initial adsorption rate was fast because Cd in the solution could quickly occupy the free active sites on the surface or inside the pores of BFA, but the number of active sites reduced significantly with the occupation of Cd, and the adsorption rate reduced until the adsorption saturation.

**Figure 7:**
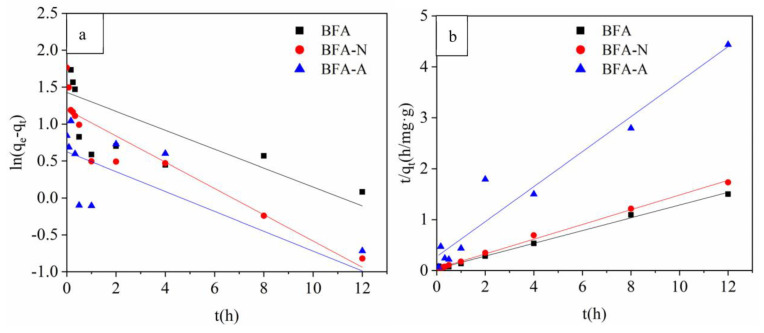
Absorption kinetic fitting of Cd by BFA, BFA-N, and BFA-A, (**a**) pseudo-first-order kinetic model, (**b**) pseudo-second-order kinetic model.

Pseudo-first-order kinetic model and pseudo-second-order kinetic model were used to fit the adsorption kinetic curve. The pseudo-first-order kinetic model often expressed the adsorption process in which the reaction rate was controlled by physical diffusion. $q_{e}$ value was needed first, but as the adsorption balance needed for a long time, it was difficult to accurately measure the $q_{e}$ value, so the pseudo-first-order kinetic model was often applied only to the initial stage of adsorption kinetic, and could not accurately describe the whole adsorption process [43]. Additionally, the results (Figure 7a and Table 2) showed that the fitting effects of BFA, BFA-N, and BFA-A adsorption on Cd were poor. The adsorption processes were more consistent with the pseudo-second-order kinetic model (Figure 7b and Table 2), which indicated that the adsorption processes were mainly controlled by chemical action rather than by the physical transport step [44]. According to the $q_{e}$ values obtained from the pseudo-second-order model, the adsorption capacity could be ranked as: BFA > BFA-N > BFA-A.

#### 3.2.2. Isothermal Adsorption Experiment

Langmuir:

(3) $$\frac{C_{e}}{Q_{e}}=\frac{C_{e}}{Q_{m}}+\frac{1}{Q_{m}b}$$

Freundlich:

(4) $$\ln Q_{e}=\frac{1}{n}\ln C_{e}+\ln K_{f}$$

In Equation (3), $C_{e}$ was the equilibrium concentration, mg/L, $Q_{e}$ was the equilibrium adsorption capacity, mg/g, $Q_{m}$ was the maximum adsorption capacity, mg/g, b was the parameter of affinity between adsorbent and adsorbate, L/mg. In Equation (4), $K_{f}$ was the adsorption coefficient, n was the adsorption index [45].

**Figure 8:**
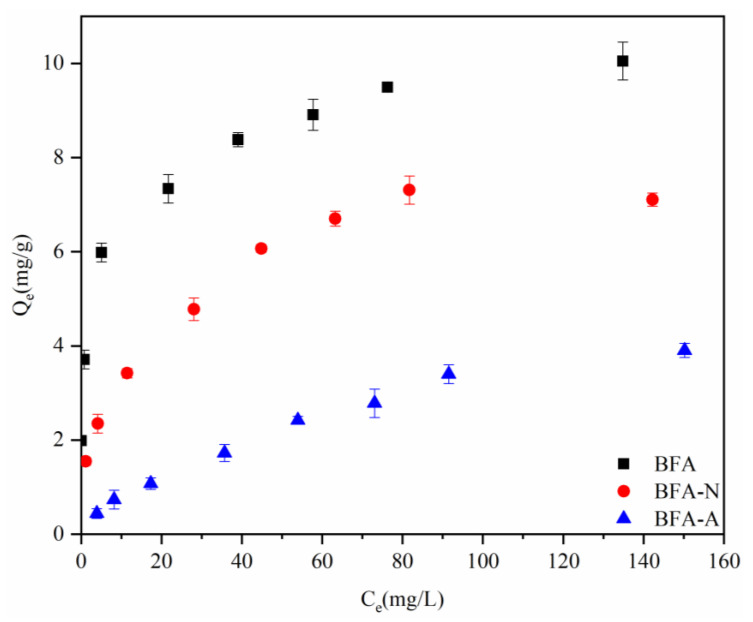
Isothermal adsorption curves of Cd by BFA, BFA-N, and BFA-A.

**Figure 9:**
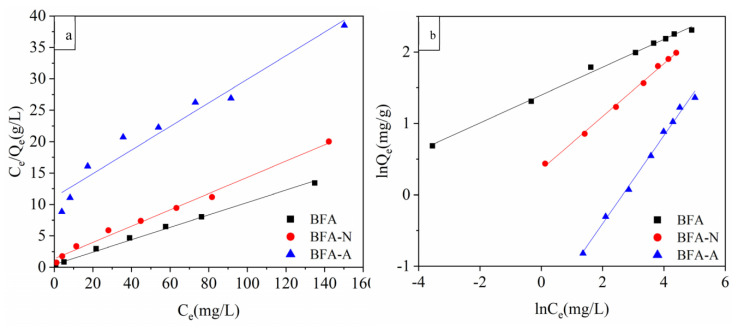
Isothermal adsorption fitting of Cd by BFA, BFA-N, and BFA-A, (**a**) Langmuir, (**b**) Freundlich.

Isothermal adsorption experiments of Cd by BFA, BFA-N, and BFA-A before and after aging were carried out. The isothermal adsorption curves were Figure 8. Both Langmuir and Freundlich equations fit the isothermal adsorption process well (Figure 9 and Table 3). According to $Q_{m}$ in Langmuir equation, the adsorption properties could be ranked as follows: BFA > BFA-N > BFA-A, which was the same as $q_{e}$ obtained from the pseudo-second-order kinetic model, but $Q_{m}$ values were generally higher than $q_{e}$.

#### 3.2.3. Precipitation Experiment

**Figure 10:**
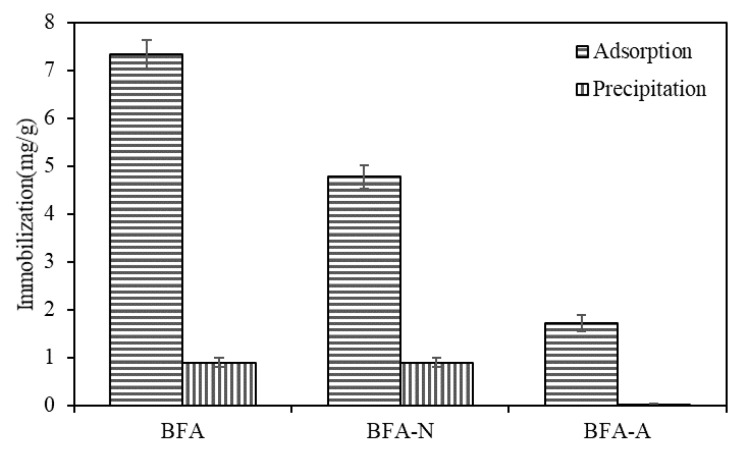
The precipitation in Cd immobilization.

In addition to the adsorption of Cd by BFA, BFA-N, and BFA-A, the adsorption capacity obtained in the isothermal adsorption experiment should also include the effect of regulating the pH of the solution by the adsorbent and causing Cd precipitation. Figure 10 revealed the precipitation when the pH of the solution was adjusted to 6.5, 6.5, and 3.7, corresponding to BFA, BFA-N, and BFA-A adsorption equilibrium, respectively. It could be concluded that the proportion of precipitation in immobilization is 12.3%, 18.8%, and 1.7%, respectively. It followed that the precipitation resulting from the change in solution pH caused by the adsorbent had some effect on Cd immobilization, but it was not the dominant one. In the soil solution system, Cd concentration was mainly controlled by adsorption and precipitation. When the Cd concentration was 10 mg/L, the pH corresponding to the equivalent point of adsorption and precipitation was about 8.5 [46]. Under acidic conditions, adsorption played a leading role. The precipitation mechanism occurred more in alkaline or Cd severely polluted soil. 

### 3.3. Influence on Cd Immobilization Mechanism Induced by the Aging of BFA 

**Figure 11:**
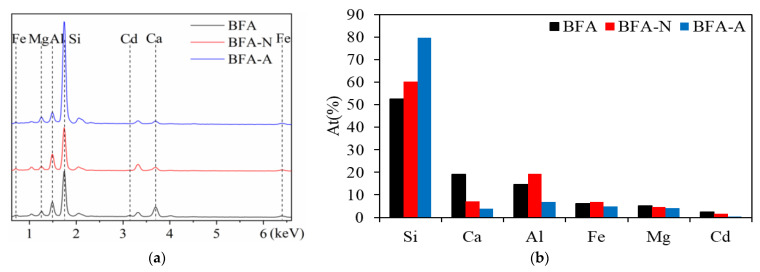
EDS of BFA, BFA-N, and BFA-A after adsorbing Cd, (**a**) energy spectrum diagram, (**b**) ratio of the elements.

**Figure 12:**
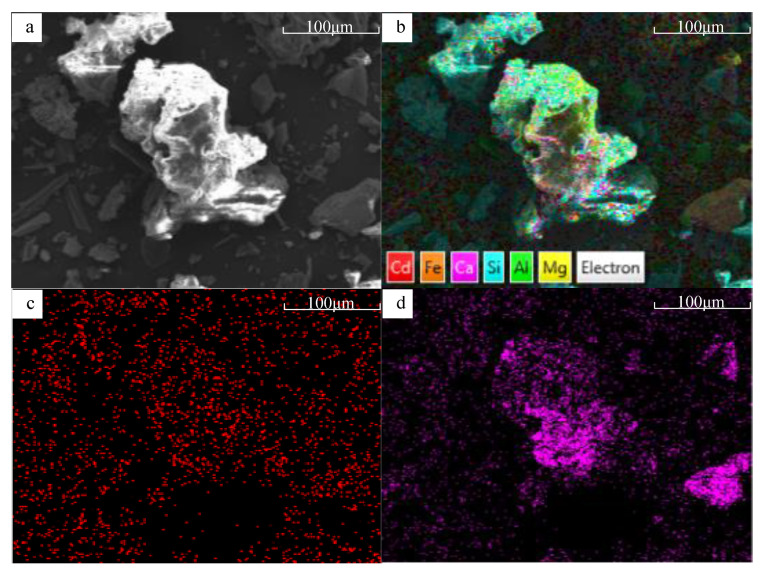
SEM of BFA-N after adsorbing Cd, (**a**) electron image, (**b**) EDS layered image, (**c**) Cd, (**d**) Ca.

**Figure 13:**
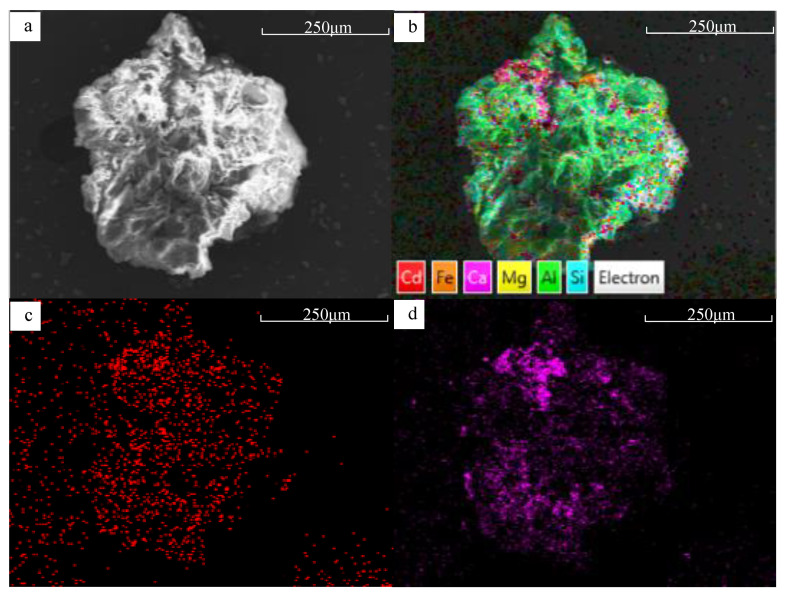
SEM of BFA-A after adsorbing Cd, (**a**) electron image, (**b**) EDS layered image, (**c**) Cd, (**d**) Ca.

Cd could be detected in BFA, BFA-N, and BFA-A after the Cd adsorption experiment (Figure 11a). As shown in Figure 11b, compared with BFA, both BFA-N and BFA-A showed Ca loss, BFA-A was more obvious than BFA-N, and BFA-A increased Al loss more than BFA-N. The Si ratio in BFA-N and BFA-A increased, there was no introduction of exogenous Si, but because of the loss of Ca and Al, the Si ratio was relatively increased, and the increase was more obvious in BFA-A. The ratios of Fe and Mg were little different. According to the SEMs of each element in BFA particles (Figure S1) before aging and BFA-N particles (Figure 12 and Figure S2) after aging, the image distribution of Ca was the most consistent with Cd among the major elements, Si, Al, Mg, Ca, and Fe. Therefore, it could be concluded that for BFA and BFA-N, the main mechanism of Cd immobilization was related to Ca, and there was no significant change. Similarly, Maria [47] tested and analyzed the aged biochar by synchrotron radiation XAFS, and found that the main mechanism of Cd immobilization was the same as that before aging, which was still CdCO_3_ formation. However, in the SEM of BFA-A, none of the distribution images of all elements, including Ca, were consistent with Cd (Figure 13 and Figure S3). Combined with the aforementioned physical and chemical properties of BFA-A, the O-C-O bond of CO_3_^2−^ in the FTIR spectrum was significantly destroyed and the pH decreased. These lead to Ca loss and the adsorption capacity of BFA-A to Cd reducing obviously. Among BFA, BFA-N, and BFA-A, the Ca content levels in Figure 11b were consistent with the Cd adsorption levels, which were shown by $q_{e}$ in Table 1, obtained by pseudo-second-order kinetic equation fitting, and $Q_{m}$ in Table 2, obtained by Langmuir equation fitting. It could be inferred that the main Cd immobilization mechanism of BFA before and after aging was consistent, and was closely related to Ca, and it might be the adsorption and precipitation of Cd on the surface of oxides, hydroxides, and salts of Ca. However, the specific mechanism between Cd and Ca still needed to be further explored by other means, such as synchrotron radiation XAFS. In addition, the Cd immobilization mechanism of BFA was found to include ion exchange, precipitation, complexation [48], obligate adsorption [49], isomorphous replacement, and others in prior studies [20]. Compared with BFA, the change of Cd immobilization mechanism of BFA-N and BFA-A after aging included: (1) the change in Zeta potential, which showed that the electrostatic adsorption of BFA-N strengthened, and the electrostatic adsorption of BFA-A weakened. (2) The amorphous state of the geopolymers in BFA-N and BFA-A could both enhance ion exchange adsorption [50,51]. (3) The change in FTIR showed that the hydroxyl complexation of BFA-N was weakened, while the hydroxyl complexation of BFA-A was enhanced.

## 4. Conclusions

Biofuel ash (BFA) was naturally aged into BFA-Natural aging (BFA-N) in the soil environment of southern China, and to simulate BFA-N, BFA was also artificially acid aged into BFA-Acid aging (BFA-A). The results indicated that there were similarities and differences in physicochemical properties between BFA-N and BFA-A, that is, the artificial acid aging method could partially simulate natural aging. The adsorption processes of BFA, BFA-N, and BFA-A were more consistent with the pseudo-second-order kinetic model compared with the pseudo-first-order kinetic model, which indicated that the adsorption processes were mainly controlled by chemical action rather than by physical transport. Both Langmuir and Freundlich equations fit the isothermal adsorption processes well. According to $Q_{m}$ in Langmuir equation and $q_{e}$ in the pseudo-second-order kinetic model, the Cd adsorption capacity of BFA reduced after natural aging, and the decrease was more obvious in BFA-A. The immobilization of Cd included adsorption and precipitation, and adsorption was the dominant factor; the precipitation proportion was only 12.3%, 18.8%, and 1.7% of BFA, BFA-N, and BFA-A, respectively. Compared with BFA, both BFA-N and BFA-A showed Ca loss, BFA-A was more obvious than BFA-N. The Ca content level was consistent with Cd adsorption level among BFA, BFA-N, and BFA-A. It could be inferred that the main immobilization mechanism of Cd by BFA before and after aging was consistent with and closely related to Ca. It might be the adsorption and precipitation of Cd on the surface of oxides, hydroxides, and salts of Ca which still need to be explored further. However, the adsorption mechanism of electrostatic interaction, ion exchange, and hydroxyl complexation changed to varying degrees in BFA-N and BFA-A.

## Figures and Tables

**Table 1:** Main element composition of BFA.

Element	SiO_2_	CaO	Al_2_O_3_	Fe_2_O_3_	K_2_O	MgO	SO_3_	Na_2_O	P_2_O_5_
Percentage (%)	47.26	19.19	12.67	7.49	4.20	3.86	1.41	1.19	1.07

**Table 2:** Absorption kinetic fitting parameters of Cd by BFA, BFA-N, and BFA-A.

	Pseudo-First-Order Kinetic Equation			Pseudo-Second-Order Kinetic Equation		
	$q_{e}$ (mg/g)	$k_{1}$ (1/h)	R^2^	$q_{e}$ (mg/g)	$k_{2}$ (g/mg·h)	R^2^
BFA	4.17	0.13	0.53	7.98	0.47	0.99
BFA-N	3.31	0.18	0.85	6.92	0.55	0.99
BFA-A	1.87	0.14	0.53	2.91	0.43	0.95

**Table 3:** Isothermal adsorption fitting parameters of Cd by BFA, BFA-N, and BFA-A.

	Langmuir			Freundlich		
	$Q_{m}$ (mg/g)	b (L/mg)	R^2^	$K_{f}$	n	R^2^
BFA	10.11	0.23	0.98	4.05	5.12	0.99
BFA-N	7.71	0.09	0.97	1.43	2.70	0.99
BFA-A	5.32	0.02	0.95	0.19	1.62	0.99

## Data Availability

The datasets generated and/or analyzed during the current study are not publicly available.

## Supplementary Materials

The following supporting information can be downloaded at: https://www.mdpi.com/article/10.3390/ijerph20054635/s1. Figure S1. SEM of BFA after adsorbing Cd, (a) electron Image, (b) EDS layered image, (c) Cd, (d) Ca, (e) Si, (f) Al, (g) Fe, (h) Mg. Figure S2. SEM of BFA-N after adsorbing Cd, (a) Si, (b) Al, (c) Fe, (d) Mg. Figure S3. SEM of BFA-A after adsorbing Cd, (a) Si, (b) Al, (c) Fe, (d) Mg.
