# Supplementary material for: Biofuel Ash Aging in Acidic Environment and Its Influence on Cd Immobilization
Source: Int J Environ Res Public Health. 2023 Mar 6;20(5):4635. doi: 10.3390/ijerph20054635 (PMC10002395; doi:10.3390/ijerph20054635)
Supplement: Supplementary file 1 [file ijerph-20-04635-s001.zip › ijerph-2266031-supplementary.pdf]

Supplementary data for

Biofuel Ash Aging in Acidic Environment and Its Influence on Cd Immobilization

Le Song [1,2,3], Feng Zhao [4], Haiyang Cui [5,6], Jingmin Wan [3] and Hui Li [1,3,*]

[1] Hebei and China Geological Survey key Laboratory of Groundwater Remediation, Institute of Hydrogeology and Environmental Geology, Chinese Academy of Geological Sciences, Shijiazhuang 050061, China
[2] School of Resources and Environmental Engineering, Hefei University of Technology, Hefei 230009, China
[3] Hebei Province Collaborative Innovation Center for Sustainable Utilization of Water Resources and Optimization of Industrial Structure, Hebei GEO University, Shijiazhuang 050031, China
[4] Hebei Geological Environment Monitoring Institute, Shijiazhuang 050021, China
[5] College of Home Economics, Hebei Normal University, Shijiazhuang 050024, China
[6] Shijiazhuang City Longquan Lake Garden Affairs Center, Shijiazhuang 050000, China
* Correspondence: lihui@mail.cgs.gov.cn

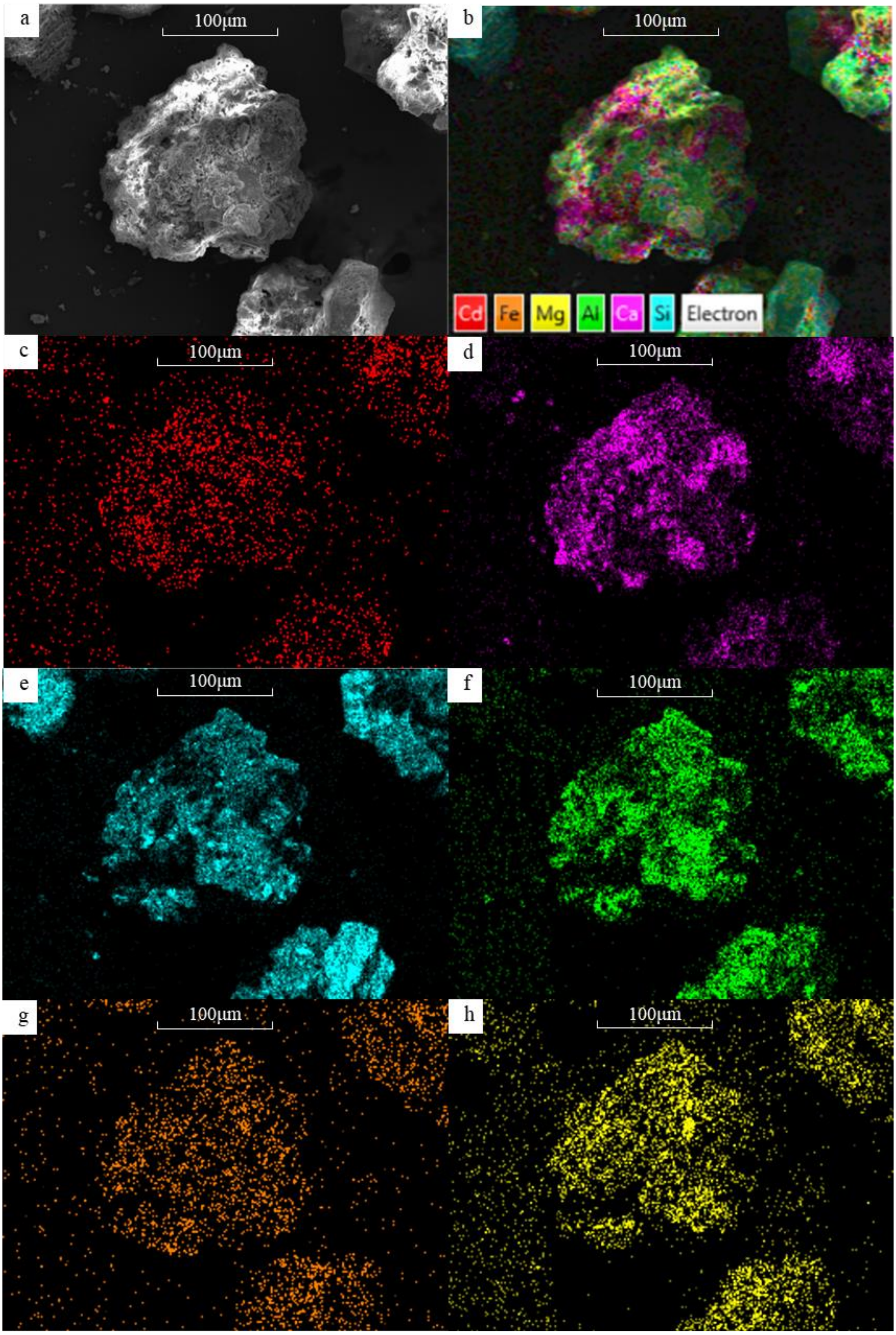

Figure S1. SEM of BFA after adsorbing Cd, (a) electron Image, (b) EDS layered image, (c) Cd, (d) Ca, (e) Si, (f) Al, (g) Fe, (h) Mg.

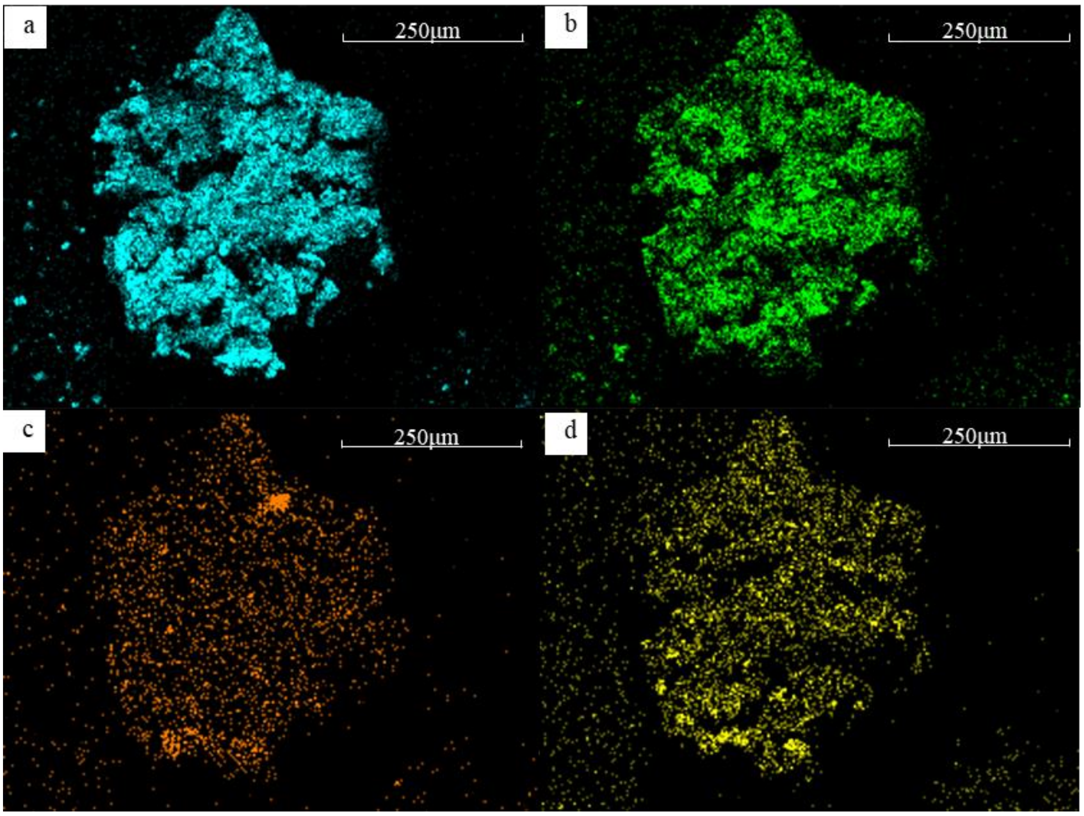

Figure S2. SEM of BFA-N after adsorbing Cd, (a) Si, (b) Al, (c) Fe, (d) Mg.

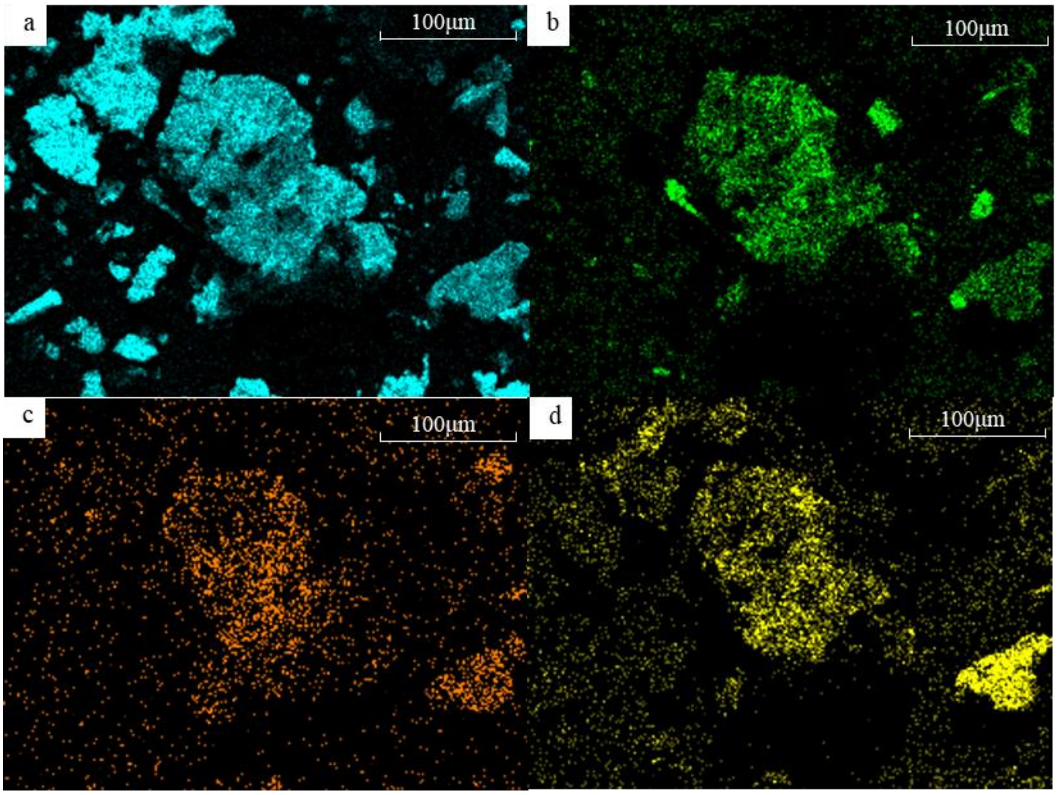

Figure S3. SEM of BFA-A after adsorbing Cd, (a) Si, (b) Al, (c) Fe, (d) Mg.
